# Supplementary material for: The propensity to sign-track is associated with externalizing behavior and distinct patterns of reward-related brain activation in youth
Source: Sci Rep. 2023 Mar 16;13:4402. doi: 10.1038/s41598-023-30906-3 (PMC10020483; doi:10.1038/s41598-023-30906-3)
Supplement: Supplementary file 4 — Supplementary Information 2. [file 41598_2023_30906_MOESM4_ESM.pdf]

Video 1: Illustration of Sign-Tracking Behaviors

A 39s video clip (.mp4 format) displaying the sign- and goal-tracking paradigm and an example response from a participant demonstrating sign-tracking behaviors. The video features two trials out of 10 in a block. For each trial, the lever extends and retracts from the response box to the right of the participant and the reward is subsequently dispensed from the response box to the left of the participant independent of participant engagement. In each trial, the participant in this video waits for the lever cue, engages with the lever, and retrieves the reward. Caregivers provided informed consent for publication of video/image material.

Video 2: Example of Non-Sign-Tracking Behaviors

A 39s video clip (.mp4 format) displaying the sign- and goal-tracking paradigm and an example response from a participant demonstrating non-sign-tracking behaviors. The video features two trials out of 10 in a block. For each trial, the lever extends and retracts from the response box to the right of the participant and the reward is subsequently dispensed from the response box to the left of the participant independent of participant engagement. The participant in this video initially attends to but does not interact with the cue, then focuses attention elsewhere and retrieves the reward each time it is dispensed. Caregivers provided informed consent for publication of video/image material.
